# Use of diagnostic tests and the appropriateness of the treatment decision in patients with suspected urinary tract infection in primary care in Denmark – observational study

**DOI:** 10.1186/s12875-018-0754-1

**Published:** 2018-05-16

**Authors:** Gloria Córdoba, Anne Holm, Tina Møller Sørensen, Volkert Siersma, Håkon Sandholdt, Marjukka Makela, Niels Frimodt-Møller, Lars Bjerrum

**Affiliations:** 1 The Research Unit for General Practice and Section of General Practice, Department of Public Health, University of Copenhagen, Øster Farimagsgade 5, 1014 Copenhagen, Denmark; 2 Department of Veterinary Clinical Sciences, University of Copenhagen, Copenhagen, Denmark; 3 Finnish Office for Health Technology Assessment (FINOHTA) - National Institute for Health and Welfare, Helsinki, Finland; 4 Department of Clinical Microbiology, Rigshospitalet, Copenhagen, Denmark

**Keywords:** Treatment decision, Point-of-care systems, Urinary tract infections, Anti- bacterial agents, Primary care, Diagnostic test

## Abstract

**Background:**

Inappropriate prescription of antibiotics is the leading driver of antimicrobial resistance (AMR). The majority of antibiotics are prescribed in primary care.

Understanding how general practitioners (GPs) use diagnostic tests and the effect on treatment decision under daily practice conditions is important to reduce inappropriate prescription of antibiotics. The aim of the study was to investigate the use of diagnostic tests in primary care patients with suspected urinary tract infection (UTI) and to assess the appropriateness of the treatment decision (TD) under daily practice conditions in Denmark.

**Methods:**

Prospective observational study. Symptomatic adult patients consulting general practice with suspected UTI recruited over 12 months. The diagnostic workup was registered in a standardized form. The appropriateness of the TD was assessed based on the results of a culture performed at a reference microbiological laboratory. TD was considered appropriate if a patient had a positive culture and was prescribed antibiotics or had a negative culture and was not prescribed antibiotics. TD was considered inappropriate if a patient had a negative culture and was prescribed antibiotics (overtreatment) or had a positive culture and was not prescribed antibiotics (undertreatment).

**Results:**

Four hundred and eighty-eight patients were included. Dipstick was used in 98% of the patients and urine culture was used in 89% of the patients; 317 had the culture performed in practice and 117 had the culture performed at the hospital. The appropriateness of the final TD was significantly (*p* = 0.04) lower in patients without culture (55%) than in patients with culture performed in practice (71%) or at hospital (69%).

**Conclusion:**

In a context with wide availability of diagnostic tests, GPs use diagnostic tests for the decision-making process in all patients with suspected UTI. Urine culture is used in the majority of the patients and is associated with a higher proportion of appropriate treatment decisions. Performance of urine culture is therefore important in reducing inappropriate antibiotic prescribing in patients with suspected UTI seeking care in general practice in Denmark.

**Trial registration:**

ClinicalTrials.gov NCT02249273.

**Electronic supplementary material:**

The online version of this article (10.1186/s12875-018-0754-1) contains supplementary material, which is available to authorized users.

## Background

Optimizing the use of antimicrobials and increasing the investment in diagnostic tests are part of the key strategies of the World Health Organization (WHO) for counteracting the spread and development of antimicrobial resistance (AMR) [1].

Denmark has a low prescription rate of antimicrobials at primary health care level in comparison to other European countries [2]. Nonetheless, there is still a high use of antibiotics at primary health care level [3].

In Denmark, suspected Urinary tract infection (UTI) is a frequent cause for antibiotic prescribing [4] and use of point-of-care tests (POCTs) [5]. There are no national guidelines on the diagnostic procedures to be followed in patients with suspected UTI. It is up to the GPs as managers/owners of the practice to decide the type of urine tests that are available in their practice. Furthermore, it is up to the GPs as clinicians to decide whether a diagnostic test should be performed in a patient with suspected UTI.

There is wide availability of diagnostic tests, which can be used in patients with suspected UTI. The diagnostic workup may include 1) dipstick, 2) microscopy, 3) culture performed and interpreted in practice, and/or 4) culture sent to a hospital laboratory. All practices can send urine samples for culture at hospital and most practices have a small laboratory where they can perform simple testing. GPs receive a fee for performing tests in practice, and there is no economic restriction to the number of tests per individual patient.

The use of urine culture implies a two-step treatment decision: first a decision when the patient consults the GP (index consultation), and a second step after the result of the urine culture is available. For cultures performed in practice, the results can be seen 1 day after the index consultation, while it may take up to 3 or 4 days to receive the results from a hospital.

In busy primary care, diagnostic test results may not translate directly to treatment decisions [6, 7], as these decisions are affected by several contextual factors [8, 9]. Therefore, the impact of diagnostic workup on appropriate treatment should be assessed under daily practice conditions.

The aim of this study was to describe the use of diagnostic tests in patients with suspected UTI and to assess the impact of the diagnostic work-up on the treatment decision under daily practice conditions in Denmark.

## Methods

### Design and setting

The study protocol has been published previously [10]. An observational study of the management of patients with suspected UTI in general practice under daily practice conditions was carried out between December 2014 and December 2015. It was part of the context assessment of a quality improvement program aimed at reducing the inappropriate prescription of antibiotics in patients with suspected UTI.

### Study population and data collection

A random sample of 500 practices from the capital region of Copenhagen was invited by letter to participate; 39 accepted the invitation. Each practice included up to 20 patients fulfilling four criteria: a) > 18 years of age, b) presence of dysuria and/or frequency, c) suspected UTI, d) not currently taking antibiotics. In a standardized form the GPs registered number of days with symptoms, types of symptoms, potential risk factors for UTI, diagnostic workup performed, and treatment decision.

### Urine sample – Reference standard

All patients were requested to provide a mid-stream urine sample (approx. 20 ml) of which half could be used for diagnostic purposes in practice, and the other half (reference standard) was cultured at a reference laboratory (Statens Serum Institute, SSI). The sample analysed by SSI was preserved in boric acid and sent by certified post on the day of the consultation. Results from the reference standard analysis were not available for the participating GPs.

At SSI, cultures were analyzed by a microbiology technician who had no information about the clinical history of the patient. A positive culture was defined as growth of ≥10^3^ colony forming units per milliliter (CFU/ml) for *E. coli* and *S. saprophyticus*, ≥10^4^ CFU/mL for other typical uropathogens and ≥ 10^5^ CFU/ml for possible uropathogens according to the European Urinalysis Standards [11].

### Definition of variables

As the main focus of analysis was the role of diagnostic tests in the decision-making process, treatment decision was selected as the primary outcome [10]. The appropriateness of the treatment decision was evaluated against the reference standard (i.e. culture at SSI). Treatment decision was considered as appropriate when a patient with a positive reference culture was prescribed antibiotics or a patient with a negative culture received no antibiotics. Treatment decision was considered as inappropriate for patients with a negative culture who received antibiotics (overtreatment) or patients with a positive culture who were not prescribed antibiotics (undertreatment).

UTI was defined as complicated if the patient was a man, a pregnant woman, a woman ≥65 years, had a relevant co-morbidity or was assessed by the nurse or GP to have complicated cystitis or pyelonephritis. An anamnestic score was calculated. It is the sum of positive findings recorded by the GP, in the standardized data collection form, during the anamnestic phase of the consultation (range 1–11).

The diagnostic pathways were categorized into three groups: a) no urine culture performed, b) urine culture performed in practice, and c) urine culture performed at the hospital.

### Statistical analysis

The statistical analysis was performed in two steps. First, possible determinants for the use of the various diagnostic tests were investigated in logistic regression models. We tested the following predictor variables individually with practices as a random intercept. The predictors were: a) anamnestic information, b) availability of diagnostic tests and c) information from any diagnostic tests previously performed (See Additional file 1: Table S1).

Secondly, we used a permutation test to assess the impact of the diagnostic pathway variable on the treatment decision . The multinomial regression model was adjusted for the predictors that were significant in the first model and the differences in baseline characteristics of the diagnostic pathway groups. All analyses were performed in the R programming language and environment v3.3.2 “Sincere Pumpkin Patch” using the lme4 and nnet package [12].

### Ethical aspects

The study was approved by the ethical committee of the Capital region (case number: H-4- 2014-097). Informed written consent was obtained from all patients participating in the study.

## Results

### Diagnostic workup

Four hundred and eighty-eight patients were recruited to the study. Dipstick was done in 480 (98%) patients and microscopy in 160 (32%) patients. 434 (89%) patients had a urine culture performed as part of the diagnostic work-up; done in practices in 317 (65%) and sent to hospital in 117 (24%) patients. The most common combination of diagnostic tests was dipstick and culture done in practice (*n* = 240, 49% of the patients) – Table 1.

**Table 1:** Distribution of the use of diagnostic tools and diagnostic pathways

	Total (*n* = 488)
Dipstick	480
Microscopy	160
Culture in practice	317
Culture sent to the hospital	117
Diagnostic pathways	
No urine culture (*n* = 54)	
Dipsticks	14
Microscopy	3
Dipsticks + microscopy	37
Urine culture in practice (*n* = 317)	
Dipsticks + urine culture in practice	240
Dipsticks + microscopy + urine culture in practice	76
Urine culture in practice	1
Urine culture sent to the hospital (*n* = 117)	
Dipsticks + urine culture in hospital	73
Microscopy + urine culture in hospital	4
Dipsticks + microscopy + urine culture in hospital	40

Dipstick results (*n* = 480) included reactions for nitrite and leucocytes. In 334 (69%) patients, the two reactions pointed in different directions: most frequently, the reaction for leucocytes was positive and for nitrite negative. 63 (13%) patients had negative reactions for both nitrite and leucocytes and 91 (18%) were positive for both.

Microscopy results (*n* = 160) included identification of ≥ 1 bacteria per High Power Field (HPF) in 106 (66%) patients; and identification of ≥ 1 white blood cell per HPF in 70 (43%) patients. Combination of ≥ 1 bacteria + ≥ 1 white blood cell per HPF in 30 (19%) patients.

The sensitivity of urine culture performed and interpreted in practice was 94% (95% CI 89%; 96%), specificity 51% (95%CI 43%; 59%), Positive Predictive Value – PPV 69% (95% CI 63%; 74%), Negative Predictive Value – NPV 88% (95% CI 79%;93%) – see Additional file 1: Table S2.

The sensitivity of urine culture performed at hospital was 90% (95% CI 80%; 95%), specificity 89% (95% CI 78%;94%), PPV 90% (95% CI 80%;95%), NPV 89% (95% CI 78%;94%) – see Additional file 1: Table S3.

Table 2 shows the distribution of baseline characteristics in patients without culture, those where culture was done in practices and patients whose culture was sent to hospital. The proportion of uncomplicated UTIs was higher (70%) in patients with no culture performed than in patients with practice culture (60%) or hospital culture (34%).

**Table 2:** Patients’ baseline characteristics and diagnostic pathways

	No culture (n = 54)	Culture performed in practice (n = 317)	Culture performed at hospital (n = 117)
Prevalence confirmed UTI	29(54)	170(46)	62(52)
Uncomplicated UTI	38(70)	191(60)	41(34)
Women	51(94)	293(92)	110(94)
Younger patients (<=64 years)	41(76)	218(69)	88(75)
Number of days with symptoms (Median-IQR)	2(1;4)	2(1;5)	2(1;4)
Signs & Symptoms			
Dysuria	38(70)	214(67)	88(75)
Frequency	38(70)	211(66)	84(72)
Urgency	24(44)	152(48)	73(62)
Suprapubic pain	17(31)	85(27)	45(38)
Dysuria and frequency	25(46)	149(47)	62(52)
Dysuria and urgency	17(31)	106(33)	57(49)
Dysuria and suprapubic pain	11(20)	57(18)	32(27)
Flank pain	4(7)	53(17)	17(14)
Genital symptoms	4(7)	30(9)	16(13)
Reported Fever	1(2)	27(8)	9(7)
Offensive smell	6(11)	57(18)	26(22)
Macrohematuria	4(7)	31(10)	17(15)
UTI within 4 weeks	0	18(6)	14(12)
Other^a^	6(11)	27(8)	15(13)
Anamnestic score^b^	2(2;3)	3(2;4)	3(2;4)

Figures are n (%), if not marked otherwise ^a^ Signs and symptoms not listed in the questionnaire

^b^Median IQR (Interquartile range) of signs, symptoms and anamnesis of previous UTI (Score 1 to 11)

Identification of predictors for use of additional diagnostic testing is shown in Additional file 1: Table S1. Neither a suspected complicated UTI nor the results of dipstick or microscopy predicted the incorporation of urine culture within the diagnostic workup. The incorporation of a diagnostic test was predicted by the availability of diagnostic tests. For example, availability of a microscope in practice decreased the propensity of performing a culture in practice (OR 0.04, 95% CI 0.004; 0.5) and availability of culture in practice decreased the propensity of performing a culture at hospital (OR 0.01, 95% CI 0.001; 0.08).

The incorporation of a diagnostic test was predicted as well by anamnestic information. For example, a high anamnestic score decreased the likelihood of performing urine culture in practice OR 0.7 (95% CI 0.6; 0.9), but increased cultures at hospital OR 1.5 (95% CI 1.1; 1.9).

Offensive smell increased urine cultures at hospital OR 2.9 (95% CI 1.2; 7), but not in practice.

### Diagnostic workup and treatment decisions

Table 3 shows the distribution of appropriate treatment decisions in the three diagnostic pathway groups. The distribution of the treatment decision was different between the three diagnostic pathways. For example, the day of the index consultation (i.e. first treatment decision), overtreatment was seen in 39% of patients without culture, 14% of those with culture in practice, and 27% of the patients with culture sent to hospital (*p*-value: 0.0001).

**Table 3:** Appropriateness of the treatment decision and use of diagnostic tests

	First treatment decision (*p*-value = < 0.0001)^a^			Final treatment decision (*p*-value = 0.04)^b^		
	Appropriate	Overtreatment	Undertreatment	Appropriate	Overtreatment	Undertreatment
No culture^c^ (n = 54)	30(55)	21(39)	3(6)	30(55)	21(39)	3(6)
Culture performed in practice (n = 317)	176(56)	46(14)	95(30)	227(71)	76(24)	14(5)
Culture performed at hospital (n = 117)	71(61)	32(27)	14(12)	81(69)	24(21)	12(10)

Appropriate = prescribing antibiotics when reference test is positive and not prescribing antibiotics when reference test is negative

Ovrertreatment = prescribing antibiotics when reference test is negative

Undertreatment = not prescribing antibiotics when reference test is positive

Permutation LR test to assess whether treatment decision at the day of the index consultation^a^ or at the day of the final treatment decision^b^ differs between groups: a) no culture, b) culture performed in practice, c) culture performed at hospital. Hierarchical model with practices as a random intercept and adjusted for: complicated versus uncomplicated UTI, offensive smell, anamnesis score, microscope and culture in practice availability

^c^When not performing culture, first treatment decision is the final treatment decision

For the final treatment decision, an appropriate treatment decision was taken in: 55% of patients without culture, 71% of those with culture in practice, and 69% of the patients with cultures sent to hospital (*p*-value = 0.04).

## Discussion

### Summary of results

In a practice setting with wide availability of diagnostic tests, nearly all patients with suspected UTI are examined with urine dipstick and for most, urine culture is part of the diagnostic workup. Neither a suspected complicated UTI nor the results of dipstick or microscopy predict the incorporation of urine culture within the diagnostic workup. Incorporation of urine culture within the decision-making process is associated with a higher proportion of appropriate treatment decisions.

### Strengths and limitations

Although a randomized controlled trial might be considered the ideal design to assess the effect of diagnostic tests on treatment decisions, there is no consensus about the best study design to help understand the link between combination of diagnostic test, test interpretation and treatment decision [6, 13, 14]. Since our study aimed at describing the use of diagnostic tests and the appropriateness of the treatment decision under daily practice conditions, a prospective observational design was a sensible way of capturing the whole decision-making process from consultation to final treatment decision [15].

The observational study was carried out as part of the context assessment of a quality improvement program. It brings about some advantages and disadvantages.

On one hand, the external validity needs to be considered with caution. Only 39 practices out of 500 accepted to participate. Previous studies have shown that GPs that participate in research or quality improvement activities tend to prescribe fewer antibiotics and are possibly more interested in reducing inappropriate prescribing than their non-participating colleagues [16, 17]. Therefore, the differences shown in this study may be conservative.

On the other hand, observation bias was minimized by the fact that GPs were interested in obtaining an accurate recording of their decisions under daily practice conditions. This type of medical audit has been extensively used in Denmark for more than 25 years [18]. The cornerstone of the methodology is the bottom-up approach in which the GPs set their own improvement goals. Hence, there is no point to record data that do not reflect their everyday practice.

Finally, we cannot completely rule out residual confounding due to unmeasured variables. Nonetheless, the effect of clustering was considered in the assessment of predictors and differences between the diagnostic groups. The model to test the association between the diagnostic pathway groups and treatment decision was adjusted by the predictors for the combination of tests and differences in baseline characteristics between the diagnostic pathway groups.

### Interpretation of the findings in relation to previous literature

This study shows the complexity of the decision-making process in a group perceived as “easy” patients in general practice [19].

International guidelines recommend the inclusion of urine culture as part of the diagnostic process only in suspected complicated UTI to target the right antibiotic choice [20, 21]. In our study, an uncomplicated versus complicated UTI did not predict the incorporation of urine culture within the diagnostic workup. It may be explained by the current debate on finding a new approach [22] to define the type of UTI based on burden of symptoms, risk factors, and availability of appropriate antimicrobial therapy. For example, in the no culture group the median anamnestic score was lower than in the other two groups. It means GPs possibly considered these patients as “uncomplicated cases”. Furthermore, the higher the anamnestic score the higher the probability to send the urine culture to the hospital and the lower the probability to perform the urine culture in the practice.

Results from the tests performed previously did not predict incorporation of a test within the diagnostic work-up. It indicates a quality problem in the use of diagnostic tests. An obvious reason for the unsystematic use of diagnostic tests might be the payment scheme. Danish primary care system provides economic incentive for the GP to perform laboratory testing in practice, and this may have influenced the diagnostic workup.

Nonetheless, shortcomings of the dipstick and microscopy may lead to the incorporation of another test in order to gain certainty on the diagnosis. The use of dipsticks in primary care needs to take into consideration two important factors for the interpretation [11]. Firstly, a minimum bladder incubation time of 4 h, what is difficult if we take into consideration that one of the main motives of consultation is frequency. Secondly, common uropathogens such as Enterococcus spp. and Staphylococcus spp. do not reduce nitrites. Microscopy requires expertise and the sensitivity is low with colony counts < 10^5^ [23]. Then, Danish GPs may rely more on the results of a urine culture.

Behavioural and medical reasons may explain the inclusion of urine culture in the diagnostic workup for most patients too.

Sackett et al. have long ago described four diagnostic approaches in clinical practice [24]. A mixture of the hypothetico-deductive strategy where the clinician gathers information to either confirm or refute his/her hypothesis and the complete history strategy could explain our results.

In a country with a strong antibiotic stewardship program, GPs are aware of the societal consequences of unnecessary use of antibiotics. They need to find a way to counteract the systematic error called “confirmation bias” [25]. This error arises from the tendency to seek and interpret evidence that predominantly confirms a pre-existing hypothesis. In our population, patients entered the study with a suspected UTI, thus for the GPs it may be difficult to withhold antibiotic prescribing. Not surprisingly, inappropriate use of antibiotics is mainly driven by overprescribing of antibiotics and only to a lesser extent by underprescribing.

Cardinal symptoms such as dysuria, frequency and urgency has a high negative predictive value [26]. It means, they are useful to rule out a UTI when absent. However, in our study population, one of the inclusion criteria was dysuria and/or frequency so the negative predictive value could not be used to rule out a UTI.

Urine culture performed in a laboratory is currently regarded as the best available test to rule in or out an infection [23]. Thus, GPs may see the use of urine culture as the best way to counteract “the confirmation bias”.

The clinical history plays a role when GPs decide whether to perform a urine culture in the practice or send it to the hospital. This has an effect as well on treatment decision.

In patients whose urine culture was performed in practice, the expectation of obtaining a result the following day combined with low anamnestic scores encouraged both GPs and patients to withhold antibiotics. The day of the index consultation, only 14% of patients were over-treated. However, incorrect interpretation of the culture resulted in 24% overtreatment for the final treatment decision.

For patients with hospital culture, a few days’ delay of culture result combined with a higher symptom score led to more antibiotic overprescribing at the day of the index consultation (27%). It is in line with a qualitative study on TDs in patients with suspected UTI, where GPs preferred to treat empirically due to the discomfort experienced by the patient [19].

### Perspectives

Implementation of effective interventions to reduce inappropriate use of antibiotics requires thorough knowledge of the context in which the decision to prescribe antibiotics is taken [27]. For example, for the group of GPs that participated in this study, education on interpretation of urine culture in practice or improving communication with the microbiology department are interventions that might reduce the inappropriate prescribing of antibiotics.

This assessment should encourage the implementation of studies to assess the use of the available diagnostic tests to set-up improvement strategies that can reduce the unnecessary prescription of antibiotics within each context/country.

Finally, the results presented in this paper focus on the impact on treatment decision. Further research assessing the cost-effectiveness and impact on patients’ outcomes should contribute with information to evaluate the multi-dimensional value of these diagnostic strategies.

## Conclusion

In a context with wide availability of diagnostic tests, GPs use diagnostic tests in all the patients with suspected UTI. It may be explained by economic as well as medical/behavioural reasons. Inclusion of urine culture as part of the diagnostic workup leads to appropriate treatment decision in the majority of the patients. Performance of urine culture is important for reducing inappropriate prescribing in patients with suspected UTI seeking care in general practice in Denmark.

## Additional file

Additional file 1: **Table S1.** Predictors for using diagnostic tests in patients with suspected UTI. Summary of the results of the logistic regression models to test the predictors for using microscopy, urine culture in practice or urine culture at the hospital. **Table S2.** Accuracy of urine culture in practice. Distribution of the interpretation of the test in relation to the reference standard. **Table S3.** Accuracy of urine culture in the hospital. Distribution of the interpretation of the test in relation to the reference standard. (DOCX 22 kb)

## Abbreviations

AMR: Antimicrobial resistance
GP: General Practitioner
NPV: Negative predictive value
PPV: Positive predictive value
SSI: Statens Serum Institute
TD: Treatment decision
UTI: Urinary tract infection
WHO: World Health Organization
