# Supplementary material for: Use of diagnostic tests and the appropriateness of the treatment decision in patients with suspected urinary tract infection in primary care in Denmark – observational study
Source: BMC Fam Pract. 2018 May 16;19:65. doi: 10.1186/s12875-018-0754-1 (PMC5956889; doi:10.1186/s12875-018-0754-1)
Supplement: Supplementary file 1 — Table S1. Predictors for using diagnostic tests in patients with suspected UTI. Summary of the results of the logistic regression models to test the predictors for using microscopy, urine culture in practice or urine culture at the hospital. Table S2. Accuracy of urine culture in practice. Distribution of the interpretation of the test in relation to the reference standard. Table S3. Accuracy of urine culture in the hospital. Distribution of the interpretation of the test in relation to the reference standard. (DOCX 22 kb) [file 12875_2018_754_MOESM1_ESM.docx]

**Table S1. Predictors for using diagnostic tests in patients with suspected UTI (N=488)**

|  |  | **Microscopy** |  | **Culture performed in practice** |  | **Culture performed at hospital** |  |
| --- | --- | --- | --- | --- | --- | --- | --- |
|  | **N** | **OR** | **CI** | **OR** | **CI** | **OR** | **CI** |
| Complicated UTI | 183 | 0.7 | 0.3;1.9 | 0.8 | 0.4;1.4 | 1.5 | 0.7;2.9 |
| Woman | 454 | 1.6 | 0.2;12 | 1.4 | 0.7;2.7 | 0.3 | 0.1;1.3 |
| <=64 years | 347 | 2 | 0.6;5.7 | 1 | 0.9;1 | 0.5 | 0.2;1.2 |
| Days with symptoms | 276 | 1.1 | 0.9;1.2 | 1 | 0.9;1 | 1 | 0.9;1 |
| **Signs and Symptoms** | | | | | | | |
| Dysuria | 340 | 1 | 0.7;1.6 | 0.6 | 0.3;1.3 | 1.1 | 0.5;2.4 |
| Frequency | 333 | 0.9 | 0.7;1.6 | 0.6 | 0.3;1.1 | 1.7 | 0.8;3.6 |
| Urgency | 249 | 0.6 | 0.2;1.6 | 0.6 | 0.3;1.1 | 2.1 | 1;4 |
| Suprapubic pain | 147 | 1.5 | 1;2.2 | 0.7 | 0.3;1.3 | 1.4 | 0.6;2.8 |
| Flank pain | 74 | 0.7 | 0.4;1.3 | 1 | 0.4;2.2 | 1.1 | 0.4;2.7 |
| Genital symptoms | 50 | 1 | 0.5;2 | 0.9 | 0.3;2.6 | 1.2 | 0.4;3.3 |
| Reports fever | 37 | 0.8 | 0.4;1.8 | 1.3 | 0.4;4.4 | 1.8 | 0.5;5.5 |
| Offensive smell | 89 | 0.7 | 0.4;1.2 | 0.4 | 0.2;1 | **2.9** | **1.2;7** |
| Macrohematuria | 52 | 0.5 | 0.2;1 | 1 | 0.2;1 | 1.1 | 0.4;3.3 |
| UTI within the last 4 weeks | 32 | 0.9 | 0.4;2 | 1 | 0.3;3.5 | 2.7 | 0.7;10 |
| Other | 48 | 1.1 | 0.4;1.7 | 1.1 | 0.4;3.1 | 0.9 | 0.3;2.6 |
| Dysuria AND Frequency | 236 | 0.9 | 0.6;1.4 | 0.6 | 0.3;1.1 | 1.6 | 0.8;3.1 |
| Dysuria AND Urgency | 180 | 0.7 | 0.5;1.1 | 0.5 | 0.2;1 | 1.9 | 0.9;3.9 |
| Dysuria AND Suprapubic pain | 100 | 1.3 | 0.8;2 | 0.6 | 0.3;1.3 | 1.4 | 0.6;3.1 |
| Anamnesis score | 488 | 0.9 | 0.8;1 | **0.7** | **0.6;0.9** | **1.5** | **1.1;1.9** |
| **Dipstick** | | | | | | | |
| N *and* L (+)* | 91 | 1 (REF) | ---- | 1 (REF) | ---- | 1(REF) | ---- |
| N *and* L (-)* | 63 | 0.7 | 0.3;9.6 | 2.5 | 0.8;7 | 0.5 | 0.1;1.9 |
| N *or* L (+)* | 334 | 0.7 | 0.3;3.1 | 1.4 | 0.6;3 | 0.8 | 0.6;3 |
| **Microscopy** | | | | | | | |
| Microscope available | 238 |  |  | **0.04** | **0.004;0.5** | 2.7 | 0.3;21 |
| Microscopy not done vs* | 328 |  |  | 1 (REF) | ---- | 1 (REF) | ---- |
| Microscopy (+)* | 52 |  |  | 1.3 | 0.1;2.8 | 0.6 | 0.1;2.4 |
| Microscopy (-)* | 108 |  |  | 1.8 | 0.1;1.5 | 1.4 | 0.5;4.4 |
| **Culture** | | | | | | | |
| Culture available in practice | 398 |  |  |  |  | **0.01** | **0.001;0.08** |

Univariate analysis with practices as a random intercept

N (number of cases predictor)

N(nitrites), L(leucocytes)

*ANOVA test no significant for differences within the categories of each variable

**Table S2. Accuracy of urine culture in practice**

|  | Reference test positive | Reference test negative | Total |
| --- | --- | --- | --- |
| Culture in practice positive | 160 | 71 | 231 |
| Culture in practice negative | 10 | 76 | 86 |
| Total | 170 | 147 | 317 |

**Table S3. Accuracy of urine culture in hospital**

|  | Reference test positive | Reference test negative | Total |
| --- | --- | --- | --- |
| Culture in hospital positive | 56 | 6 | 62 |
| Culture in hospital negative | 6 | 49 | 55 |
| Total | 62 | 55 | 117 |
